# Immunity to Influenza B/Yamagata‐Lineage Viruses Has Not Waned Since the Disappearance of This Virus Lineage

**DOI:** 10.1111/irv.70188

**Published:** 2025-11-16

**Authors:** Hassanein H. Abozeid, Chunyang Gu, Sanja Trifkovic, Gabriele Neumann, Yoshihiro Kawaoka

**Affiliations:** ^1^ Influenza Research Institute, Department of Pathobiological Sciences, School of Veterinary Medicine University of Wisconsin‐Madison Madison Wisconsin USA; ^2^ Department of Poultry Diseases, Faculty of Veterinary Medicine Cairo University Giza Egypt; ^3^ Division of Virology, Department of Microbiology and Immunology and International Research Center for Infectious Diseases, the Institute of Medical Science University of Tokyo Tokyo Japan; ^4^ The Research Center for Global Viral Diseases National Center for Global Health and Medicine Research Institute Tokyo Japan; ^5^ Pandemic Preparedness, Infection and Advanced Research Center University of Tokyo Tokyo Japan

**Keywords:** antibody waning, B/Yamagata lineage, influenza B virus

## Abstract

**Background:**

Influenza B viruses are important contributors to seasonal influenza epidemics. Two antigenically distinct lineages, B/Victoria and B/Yamagata, have been circulating since the late 1980s. However, the B/Yamagata lineage has not been detected in most countries since 2020, potentially resulting in waning immunity that may leave people vulnerable to B/Yamagata virus infections, should this lineage reemerge.

**Methods:**

We investigated the impact of the recent lack of B/Yamagata virus circulation on immunity in adults by analyzing the hemagglutination‐inhibition (HI) titers of serum samples (*n* = 504) collected from 2013 through 2024 against influenza B viruses circulating from 1958 to 2019.

**Results:**

Human serum HI titers to B/Yamagata viruses have not markedly declined since B/Yamagata viruses were last detected in the US in 2020. Human serum HI titers were highest against the first encountered B/Victoria‐ and B/Yamagata–lineage viruses, respectively, revealing imprinting effects.

**Conclusions:**

The recent disappearance of the B/Yamagata lineage has not led to a substantial decline in antibody levels against this lineage.

## 1 Introduction

Influenza B viruses (IBVs), first identified in the 1940s, are major contributors to seasonal influenza epidemics. Antigenic characterization of IBVs identified three major antigenic groups: ‘B/Ancestral’, ‘B/Victoria’ (named after the reference strain B/Victoria/2/1987), and ‘B/Yamagata’ (named after the reference strain B/Yamagata/16/1988) [1]. Influenza B/Ancestral viruses circulated until the early 1970s and evolved into B/Victoria‐like viruses (represented by B/Hong Kong/5/1972). From this B/Victoria‐like lineage, two distinct lineages subsequently emerged: B/Victoria and B/Yamagata [1]. The B/Yamagata lineage emerged in the late 1980s and dominated in the 1990s in most parts of the world [2, 3]. The B/Victoria lineage was mainly confined to China during the 1990s but re‐emerged globally in the early 2000s [4] and viruses of both lineages co‐circulated for the following two decades.

Both lineages exhibit unique dynamics [5]: B/Victoria viruses show a higher rate of antigenic drift than B/Yamagata viruses, leading to faster antigenic changes [5, 6]. In addition, B/Victoria viruses infect mainly younger people, whereas B/Yamagata viruses have a bimodal age distribution infecting mainly young children and old adults with a notable gap of infections in young adults [7, 8].

Immune imprinting describes a phenomenon where the first exposure to an influenza virus shapes the immune response to subsequent infections [9]. For example, Gostic et al. [10] demonstrated that first exposure to an influenza A/H1N1 virus reduces the future risk of severe infections with A/H1N1 viruses, but also A/H5N1 viruses because H1 and H5 HA belong to the same phylogenetic super‐group of HAs (group 1). Similarly, first exposure to an influenza A/H3N2 virus reduces the risk of severe infection with A/H3N2 and A/H7N9 viruses because H3 and H7 HAs belong to the same super‐group of HAs (group 2). For IBVs, imprinting has now been described as well [11, 12]. Specifically, Edler et al. [13] found the highest HI titers to B/Yamagata viruses in individuals born in the 1980s and 1990s, the highest HI titers to B/Victoria viruses in individuals born in the 1960 and 1970, and the highest HI titers to B/Ancestral viruses in individuals born before the 1960s, demonstrating that the first exposure to an IBV leaves a lifelong immunological imprint. Skowronski et al. [14] revealed that children primed with B/Yamagata vaccines recall strong immunity to B/Yamagata lineage after receiving multiple doses of B/Victoria vaccine. Vieira et al. [15] found that first infection with a B/Yamagata virus reduced the risk of future B/Yamagata virus infection, but the data were not significant for B/Victoria viruses. Moreover, B/Yamagata viruses are no longer circulating and have not been reported in the US since 2020 [16, 17, 18]. Currently, it is not known if the absence of B/Yamagata virus infections since 2020 has resulted in waning immunity to this lineage and if such waning is affected by imprinting. Here, we sought answers to these questions by comparing anti‐influenza B antibodies in human sera collected in 2013–2019 (during Yamagata virus circulation) with those in sera collected in 2022–2024, that is, 2–4 years after this lineage was last detected in the US.

## 2 Methods

### 2.1 Serum Samples

All serum samples were purchased from BioIVT (Westbury, New York, USA) and LAMPIRE Biological Laboratories (Pipersville, PA, USA). Sera were collected from adults of unknown origin with unknown influenza exposure history in the US between 2013 and 2024 (Table 1). All sera were treated with receptor‐destroying enzyme II (RDE) (Hardy Diagnostics #370013) for 18–20 h at 37°C. Then, the sera were inactivated by incubation for 45–50 min at 56°C and were then diluted in phosphate buffered saline (PBS) (Cytiva PBS GHC‐SH30256.02) to a dilution of 1:10. The sera were preadsorbed to turkey red blood cells (TRBCs) (Rockland R313) by adding 1/10 volume of washed and packed TRBCs to the sera and incubating the mixture at room temperature for 1 h with gentle shaking. The sera were removed from the TRBCs by pelleting the cells by centrifugation and pipetting the supernatant into a fresh tube.

**TABLE 1:** Number of serum samples by year of collection.

Year of serum collection	Number of sera
2013	10
2014	9
2015	20
2016	33
2017	75
2019	96
2022	83
2023	80
2024	98
Total	504

### 2.2 Viruses

Twenty‐two IBVs representing the B/Ancestral (*n* = 4), B/Victoria (*n* = 10), and B/Yamagata (*n* = 8) lineages were selected based on phylogenetic analyses and antigenic cartography [1, 19]. The B/Ancestral lineage representative viruses were as follows: B/Johannesburg/1958, B/Netherlands/1000/1962, B/Victoria/98926/1970 and B/Netherlands/1000/1967. The B/Victoria lineage representative viruses were as follows: B/Hong Kong/5/1972, B/Netherlands/1000/1974, B/Johannesburg/9/1975, B/Netherlands/1000/1976, B/Netherlands/1000/1990, B/Yokohama/2086/2003, B/Brisbane/60/2008, B/Paris/1762/2009, B/Florida/78/2015 and B/Washington/2/2019. The B/Yamagata lineage representative viruses were as follows: B/Yamagata/16/1988, B/Netherlands/580/1989, B/Singapore/11/1994, B/Chantaburi/218/2003, B/Florida/4/2006, B/Phuket/3073/2013, B/Darwin/58/2019 and B/Wisconsin/200/2020. All viruses were propagated in hCK cells (a derivative of MDCK cells with higher levels of α2,6‐linked sialic acids and lower levels of α2,3‐linked sialic acids [20]) in the presence of L‐1‐tosylamido‐2‐phenylethyl chloromethyl ketone (TPCK)‐treated trypsin. Their HA gene sequences were confirmed by Sanger sequencing.

### 2.3 Hemagglutination Assay

Hemagglutination assays (HAs) were conducted in 96‐well V‐bottom plates (CELLSTAR Tissue Culture Plates, Greiner Bio‐One 651161). PBS (50 μL) was added to each well. Then, 50 μL of virus was added to the first well two‐fold serially diluted across the row. Washed 0.5% TRBCs (50 μL) were then added to each well, and the reactions were incubated for 30 min at room temperature. The HA titer was determined by tilting the plate and counting the number of wells to the endpoint of agglutination (n). The HA titer was calculated as the reciprocal of the final dilution to provide complete hemagglutination and is presented as hemagglutinating units (HAUs). This value was used to dilute the virus to 8 HAUs in PBS. Back titrations and dilution adjustments were conducted as needed to reach 8 HAUs.

### 2.4 Hemagglutination Inhibition Assay

Hemagglutination inhibition (HI) assays were performed in accordance with the World Health Organization Global Influenza Surveillance Network, Manual for the Laboratory Diagnosis and Virological Surveillance of Influenza [21]. Briefly, in a 96‐well V‐bottom plate, 25 μL of each RDE‐treated serum sample was tested over two‐fold serial dilutions from 1:10 to 1:1280 against 25 μL of standardized IBV (8 HAUs per 50 μL). Serum‐virus mixtures were incubated for 30 min at room temperature. Then, 50 μL of washed 0.5% TRBCs were added to each well and incubated at room temperature for 1 h. The HI titer was determined by tilting the plate, counting the number of wells to the endpoint of nonagglutinated cells, and converting it to the corresponding dilution value in the serum two‐fold dilution series.

### 2.5 Statistical Analysis

Statistical analyses were applied to the log‐2 transformed values of the HI titers. Data were tested for normal distribution using the Shapiro–Wilk test. Statistical significance of the difference between HI titers before and after the circulation of B/Yamagata‐lineage viruses was calculated using the unpaired *t* test with Welch corrections. The Holm–Šídák method was used for multiple comparisons with statistical significance at *p* value < 0.05. Differences in HI titers among the B/Yamagata virus sublineages were analyzed by using a one‐way ANOVA, followed by Dunnett's multiple comparison test, with statistical significance set at *p* < 0.05. All statistical analyses were performed using GraphPad Prism 9.5.1.

## 3 Results

There has been no circulation of B/Yamagata viruses in the US since 2020, and immunity to this virus lineage may have waned. Such waning might be influenced by imprinting; for example, individuals first exposed to B/Yamagata viruses may experience less waning than individuals first exposed to B/Ancestral or B/Victoria viruses. Accordingly, we tested human sera from donors with unknown exposure history collected in the US in 2013–2019 (i.e., when Yamagata‐lineage viruses dominated; *n* = 243) and 2022–2024 (i.e., 2–4 years after the last recorded Yamagata virus isolation in the US; *n* = 261). Sera were grouped into the following three birth cohorts: people born in 1941–1971 (*n* = 179), whose first exposure should have been to a B/Ancestral virus; those born in 1972–1987 (*n* = 193), whose first exposure should have been to a B/Victoria virus; and those born in 1988–1999 (*n* = 132), whose first exposure should have been to a B/Yamagata virus. All sera were tested in HI assays against human IBVs representing B/Ancestral (isolated in 1958–1967), B/Victoria (isolated in 1968–2019), and B/Yamagata (isolated in 1988–2020) viruses, selected based on phylogenetic and antigenic analyses by Rosu et al. [1] and our group [19].

**FIGURE 1:**
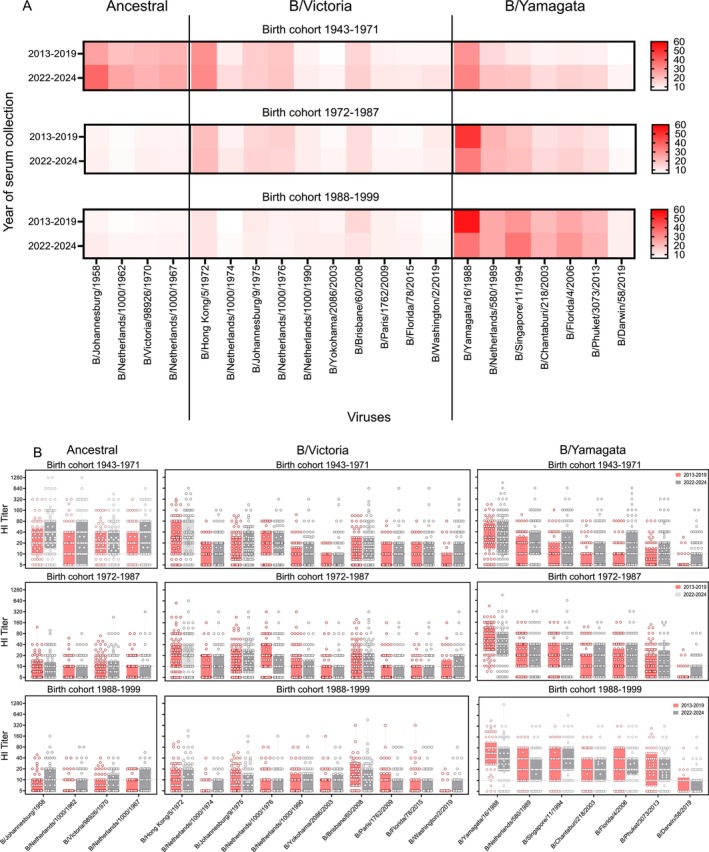
HI titers against influenza B viruses in sera from different birth cohorts collected before or after the disappearance of B/Yamagata lineage. (A) Heat map plots of the geometric mean HI titers of sera from individuals grouped by birth cohorts, collected before (2013–2019) or after (2022–2024) the disappearance of the B/Yamagata lineage. Sera were tested against the indicate influenza B viruses isolated prior to the year of serum collection. Rows show the year of serum collection; columns show the influenza B virus strains. Color intensity indicates the geometric mean of HI titers. (B) Box plots of HI titers from sera of individuals grouped by birth cohorts, collected before (2013–2019) or after (2022–2024) the disappearance of the B/Yamagata lineage. Boxes represent the interquartile range (Q1–Q3). Whiskers indicate the minimum and maximum values. Individual HI titers are represented by dots. The transverse dotted line marks the limit of detection (HI titer ≥10). Titers below 10 are plotted as 5.

First, we assessed immune responses against IBVs isolated prior to serum collection. In general, HI titers to B/Yamagata viruses were higher than those to B/Victoria viruses. B/Ancestral viruses reacted with most of the sera from individuals born between 1943 and 1971 (when the B/Ancestral viruses were circulating) (Figure 1); no significant differences were detected between sera collected in 2013–2019 and 2022–2024 (Figure 1). Individuals in the 1972–1987 and 1988–1999 birth cohorts were born after the circulation of the B/Ancestral viruses and most of their sera did not react strongly with B/Ancestral viruses, although some had measurable HI titers against these viruses (Figure 1B).

B/Victoria viruses emerged in the early 1970s, circulated in North America until the late 1980s, and then again since the early 2000s (Figure S1 and Table S1). Individuals in the 1988–1999 birth cohort, whose first IBV exposure was most likely to a B/Yamagata‐lineage virus had relatively low HI titers to B/Victoria viruses (Figure 1). Interestingly, sera from individuals whose first IBV exposure was most likely to a B/Victoria‐lineage virus (i.e., the 1972–1987 birth cohort) also had relatively low HI titers to B/Victoria viruses (Figure 1). Slightly higher HI titers to B/Victoria viruses were detected for the 1943–1971 birth cohort. Typically, the highest HI titers were detected against the earliest B/Victoria virus tested in our study (i.e., B/Hong Kong/5/72) (Figure 1).

B/Yamagata viruses emerged in the late 1980s, were dominant in the US in the 1990s, and co‐circulated with B/Victoria viruses until 2020. Consequently, most individuals in all three birth cohorts possessed serum antibodies against B/Yamagata viruses, with the exception of B/Darwin/58/2019 (Figure 1). Compared to the other birth cohorts, HI titers against B/Yamagata viruses were significantly higher for sera from the 1988–1999 birth cohort, that is, for individuals whose first IBV exposure was most likely to a B/Yamagata‐lineage virus (Figure 1). In all three birth cohorts, HI titers to the first B/Yamagata virus tested (i.e., B/Yamagata/16/1988) were higher than those against more recent B/Yamagata viruses (Figure 1). Sera collected in 2013–2019 (when B/Yamagata viruses circulated in the US) showed slightly higher HI titers to B/Yamagata/16/1988 than sera collected in 2022–2024 (i.e., 2–4 years after the last isolation of a B/Yamagata virus in the US), but these differences were not statistically significant (Figure 1).

**FIGURE 2:**
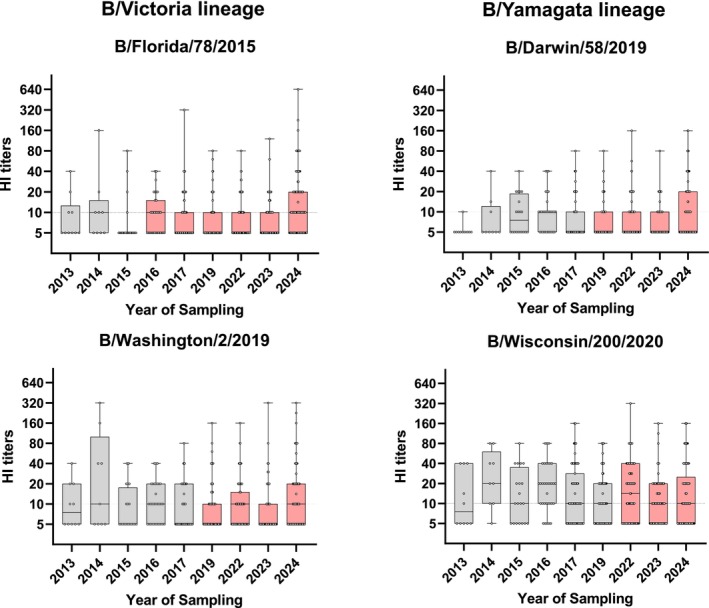
HI titers of sera collected pre– or post–virus emergence. Box plots showing the HI titers from sera of individuals grouped by birth cohort, tested against specific influenza B viruses isolated prior to (gray) or after (red) the year of serum collection. Boxes represent the interquartile range (Q1–Q3), whiskers indicate the minimum and maximum values, and individual HI titers are shown as dots. Numbers indicate the number of serum samples tested. The transverse dotted line marks the limit of detection (HI titer ≥10). Titers below 10 are plotted as 5.

We also tested the serum HI titers against viruses isolated after the sera were collected (Figure 2). Overall, we did not detect significant differences between sera collected in the year of, or prior to, virus isolation compared to sera collected in years after virus isolation, suggesting that the viruses circulating after the respective sera were collected had not undergone major antigenic changes compared to the most recent viruses tested here.

**FIGURE 3:**
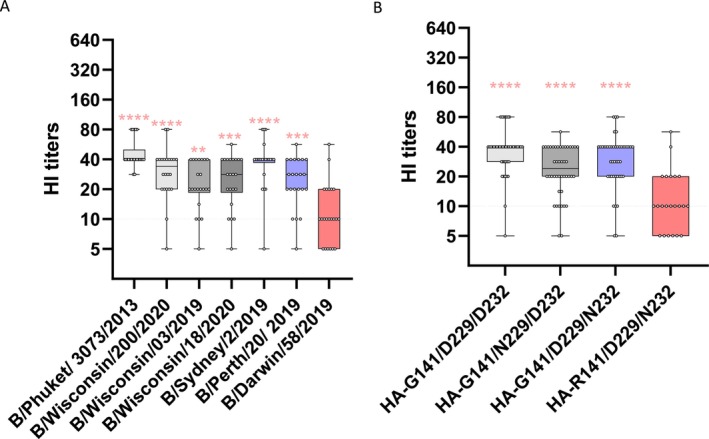
HI titers against recent B/Yamagata‐lineage viruses. (A) Box plots of HI titers from a subset of sera (*n* = 22) tested against seven viruses representing four sublineages of B/Yamagata viruses: B/Phuket/3073/2013 and B/Wisconsin/200/2020 represent the HA‐G141/D229/D323 group (light grey); B/Wisconsin/03/2019 and B/Wisconsin/18/2020 represent the HA‐G141/N229/D232 group (dark grey); B/Sydney/2/2019 and B/Perth/20/2019 represent the HA‐G141/D229/N232 group (blue); and B/Darwin/58/2019 represents the HA‐R141/D229/N232 group (red). (B) HI titers grouped by different sublineages. Boxes represent the interquartile range (Q1–Q3). Whiskers indicate the minimum and maximum values. The transverse dotted line marks the limit of detection (HI titer ≥10). Titers below 10 are plotted as 5. Statistical significance (applied on the log2‐transformed values) compared to B/Darwin/3073 (2013) or the HA‐R141/D229/N232 sublineage is represented by asterisks (**p ≤ 0.01, ***p ≤ 0.001, ****p ≤ 0.0001).

Antibodies were detected against all B/Yamagata viruses tested, with the exception of B/Darwin/58/2019, for which the HI titers were below the detection limit for most sera tested (Figure 1). Most B/Yamagata viruses circulating in 2018–2019 encoded HA‐G141/D229/D232, whereas sublineages encoded HA‐G141/N229/D232, ‐G141/D229/N232, or ‐R141/D229/N232 (including B/Darwin/58/2019) (Table 2; Figure S2); the HA‐D232N substitution creates a glycosylation site at HA‐232‐234 (NxT). To assess the consequences of these substitutions, we tested a subset of sera against representative viruses of these four sublineages (Figure 3 A,B, and Table S2). Viruses representing the HA‐G141/N229/D232 and ‐G141/D229/N232 sublineages were antigenically indistinguishable from the major lineage encoding HA‐G141/D229/D232 (Table S2). However, B/Darwin/58/2019 (encoding HA‐R141/D229/N232) did not react with most of the sera tested (Figure 3A,B and Table S2), suggesting that it is an antigenic variant that circulated in 2018–2019 to which most people had not been exposed. This loss of reactivity likely originates from the HA‐G141R substitution, based on an HA amino acid comparison of the viruses tested here.

**TABLE 2:** Comparison of amino acids at HA positions 141, 229, and 232.

Virus	HA amino acid position		
	141	229	232
B/Phuket/3073/2013	G	D	D
B/Wisconsin/200/2020	.	.	.
B/Wisconsin/03/2019	.	N	.
B/Wisconsin/18/2020	.	N	.
B/Sydney/2/2019	.	.	N
B/Perth/20/2019	.	.	N
B/Darwin/58/2019	R	.	N

## 4 Discussion

In this study, we asked whether IBV immunity in adults from different birth cohorts has waned because of the lack of B/Yamagata virus infections in the US since 2020. In general, HI titers to recent B/Yamagata viruses were not significantly lower in sera collected in 2022–2024 compared to those collected in 2013–2019 indicating a lack of substantial waning of antibody titers during the time window we tested.

Antibody levels typically peak 2–6 weeks after exposure to a virus and start to decline thereafter, before leveling off at 6–12 months after exposure. Repeated exposure to antigenically similar viruses is believed to be critical to maintaining protective antibody titers. During the SARS‐CoV‐2 pandemic in 2020–2022, social distancing and personal protective equipment including masks led to a massive reduction in human influenza A and B virus infections. However, after the measures implemented to control SARS‐CoV‐2 were lifted, the number of human influenza A and B/Victoria virus infections returned to prepandemic levels, but B/Yamagata viruses have not been detected in the US since March 2020. This provided a unique opportunity to determine whether sera collected in 2022–2024 showed significantly lower HI titers to B/Yamagata viruses compared to sera collected in 2013–2019. Longitudinal studies of influenza A virus antibody titers indicated significant decreases 3.5–7 years after exposure [22, 23]. Our data indicate that, so far, HI titers to B/Yamagata viruses have not declined significantly since they were last detected in the US, suggesting that most individuals still possess some protection against B/Yamagata viruses, should they re‐emerge.

The first exposure to an antigenically novel virus leaves a lasting imprint on the immune response and influences subsequent immune responses, a phenomenon known as immune imprinting [9]. Edler et al. [13] reported that HI titers were highest against viruses encountered early in life, rather than against recently encountered isolates. Consistent with this work, here, we detected the highest HI titers against the first virus from the B/Victoria and B/Yamagata lineages, respectively, rather than against later viruses from the respective lineage.

If B/Yamagata lineage viruses were to reemerge, cross‐reactive antibodies from B/Victoria vaccines may provide some level of protection to a population with no history of B/Yamagata vaccination or infection. A study by Kiseleva et al. [24] demonstrated that ferrets vaccinated with Victoria‐lineage viruses were better protected against challenge with Yamagata‐lineage viruses than vice versa. This suggests that the continued circulation of and vaccination with Victoria‐lineage viruses may stimulate broad immune responses that might provide protection against Yamagata‐lineage viruses. Understanding these dynamics will be crucial for future public health policies.

Interestingly, we found that most sera lacked HI titers to a recent B/Yamagata virus, B/Darwin/58/2018. Further analysis revealed that this virus is antigenically different from the other viruses we tested, most likely conferred by the HA‐G141R mutation. The B/Yamagata vaccine strain (B/Phuket/3073/2013) has remained the same since 2013 and the B/Darwin/58/2018 mutant may have been the first noteworthy antigenic change in the B/Yamagata lineage in recent years. We speculate that the measures implemented in 2020 to slow the SARS‐CoV‐2 pandemic may have prevented the further spread of this antigenic variant.

This study has some limitations. First, our HI assay used whole IBV antigens rather than ether‐split preparations, which may reduce HAI sensitivity; nonetheless, all samples were processed under identical conditions, allowing comparisons across cohorts and viral lineages. Second, the vaccination history of the individuals from whom sera were collected is unknown. Because US influenza vaccines have remained quadrivalent with a B/Yamagata component throughout the period of sample collection, the lack of antibody waning may, to some extent, reflect recent vaccinations. Nevertheless, our findings on birth‐cohort–specific differences and the lasting impact of immune imprinting remain robust, as these patterns are independent of whether individuals were infected or vaccinated.

In summary, our study demonstrated that the recent lack of confirmed B/Yamagata virus infections has not yet appreciably affected the antibody levels to viruses of this lineage. Moreover, this study highlights the strong effect of early‐life immune imprinting.

## Author Contributions

**Hassanein H. Abozeid:** methodology, investigation, validation, formal analysis, visualization, writing – original draft, writing – review and editing, data curation, conceptualization. **Chunyang Gu:** methodology, investigation, validation, writing – review and editing, data curation, conceptualization, formal analysis, visualization. **Sanja Trifkovic:** writing – original draft, writing – review and editing, visualization. **Gabriele Neumann:** conceptualization, writing – original draft, writing – review and editing, project administration, supervision, visualization, methodology, formal analysis. **Yoshihiro Kawaoka:** conceptualization, writing – review and editing, project administration, funding acquisition, supervision, resources, methodology.

## Conflicts of Interest

Y.K. has received grant support from Daiichi Sankyo Pharmaceutical, Toyama Chemical, Tauns Laboratories Inc., Otsuka Pharmaceutical Co. Ltd., Shionogi & Co. Ltd., Otsuka Pharmaceutical, KM Biologics, Kyoritsu Seiyaku, Shinya Corporation, and Fuji Rebio. Y.K. and G.N. are co‐founders of FluGen. The other authors have no conflicts of interest.

## Supporting information


**Table S1:** GISAID acknowledgement.

**Table S2:** HI titers of influenza B viruses with amino acid substitutions in HA.

**Figure S1:** irv_70188‐sup‐0003‐SuppFigure1.pptx. Frequency of B/Victoria and B/Yamagata viruses from 1985 to 2003. Stacked area chart showing the relative frequency of B/Victoria‐ and B/Yamagata‐lineage viruses globally, excluding Asia, based on available HA sequences from GISAID (see Table S1) and GenBank from 1985 to 2003.

**Figure S2:** irv_70188‐sup‐0004‐SuppFigure2.pptx. Location of HA amino acid residues 141, 229 and 232 in the three‐dimensional structure. Shown is the three‐dimensional structure of influenza B/Brisbane/60/2008 HA (protein database accession #4FQM). The HA monomers are colored light blue, light gray, and dark gray. Amino acids 141, 229, and 232 are indicated in red.

## Data Availability

All data generated or analyzed during this study are available from the corresponding author upon request.
