# Supplementary figures and images for: Immunity to Influenza B/Yamagata‐Lineage Viruses Has Not Waned Since the Disappearance of This Virus Lineage
Source: Influenza Other Respir Viruses. 2025 Nov 16;19(11):e70188. doi: 10.1111/irv.70188 (PMC12620126; doi:10.1111/irv.70188)

## Slide 1
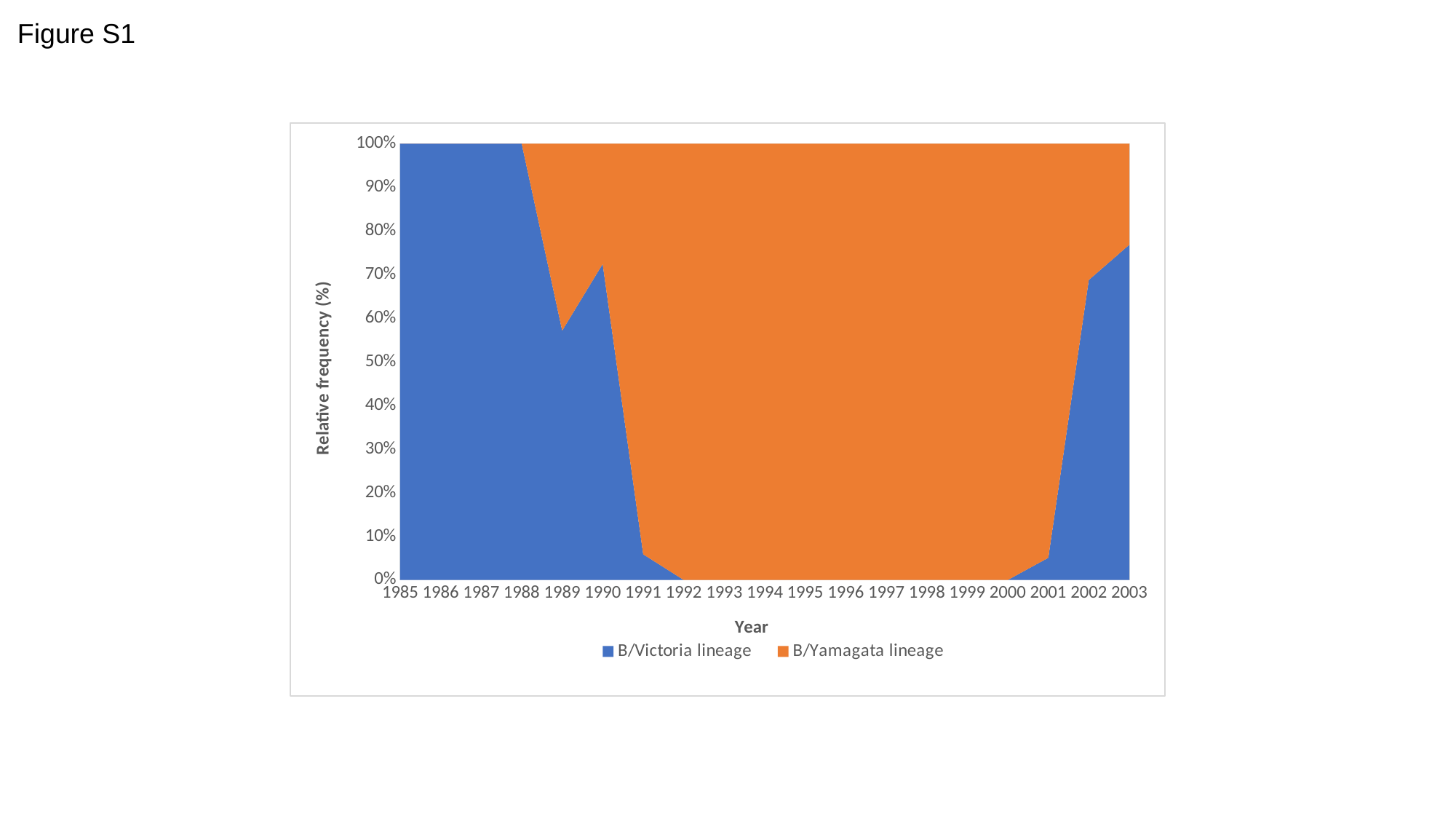

Figure S1
### Chart
| Category | B/Victoria lineage | B/Yamagata lineage |
|---|---|---|
| 1985 | 6.0 | 0.0 |
| 1986 | 5.0 | 0.0 |
| 1987 | 2.0 | 0.0 |
| 1988 | 4.0 | 0.0 |
| 1989 | 4.0 | 3.0 |
| 1990 | 21.0 | 8.0 |
| 1991 | 1.0 | 16.0 |
| 1992 | 0.0 | 7.0 |
| 1993 | 0.0 | 22.0 |
| 1994 | 0.0 | 27.0 |
| 1995 | 0.0 | 50.0 |
| 1996 | 0.0 | 28.0 |
| 1997 | 0.0 | 52.0 |
| 1998 | 0.0 | 36.0 |
| 1999 | 0.0 | 100.0 |
| 2000 | 0.0 | 88.0 |
| 2001 | 10.0 | 187.0 |
| 2002 | 231.0 | 105.0 |
| 2003 | 126.0 | 38.0 |

Supplement: Supplementary file 3 — Figure S1: irv_70188‐sup‐0003‐SuppFigure1.pptx. Frequency of B/Victoria and B/Yamagata viruses from 1985 to 2003. Stacked area chart showing the relative frequency of B/Victoria‐ and B/Yamagata‐lineage viruses globally, excluding Asia, based on available HA sequences from GISAID (see Table S1) and GenBank from 1985 to 2003. [file IRV-19-e70188-s004.pptx]

## Slide 1
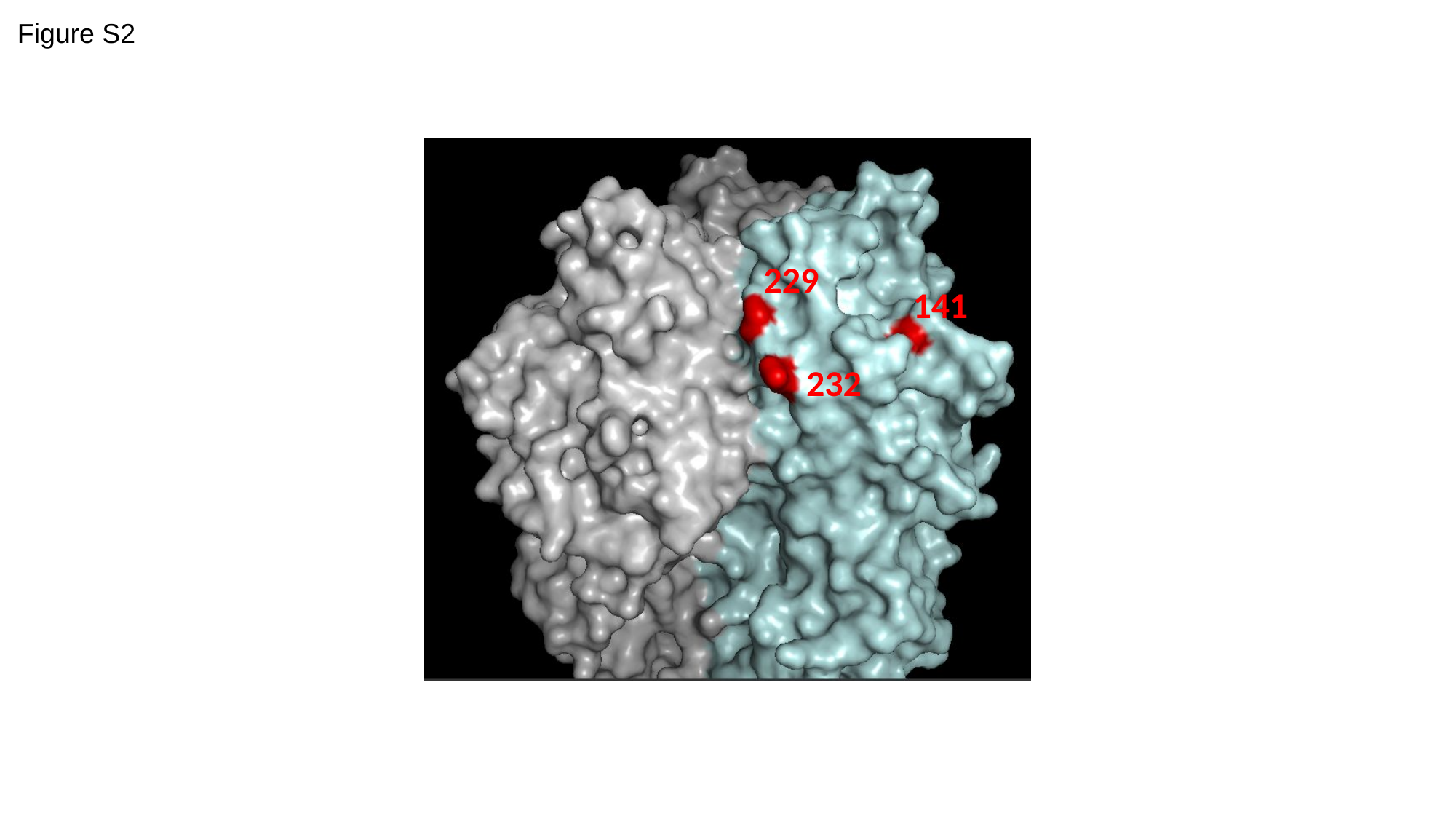

Figure S2
229
141
232

Supplement: Supplementary file 4 — Figure S2: irv_70188‐sup‐0004‐SuppFigure2.pptx. Location of HA amino acid residues 141, 229 and 232 in the three‐dimensional structure. Shown is the three‐dimensional structure of influenza B/Brisbane/60/2008 HA (protein database accession #4FQM). The HA monomers are colored light blue, light gray, and dark gray. Amino acids 141, 229, and 232 are indicated in red. [file IRV-19-e70188-s003.pptx]
